# Targeting the Hedgehog signaling pathway in cancer: beyond Smoothened

**DOI:** 10.18632/oncotarget.4224

**Published:** 2015-05-22

**Authors:** Annelies Gonnissen, Sofie Isebaert, Karin Haustermans

**Affiliations:** ^1^ University of Leuven (KU Leuven), Department of Oncology, Laboratory of Experimental Radiotherapy, Leuven, Belgium; ^2^ University Hospitals Leuven, Department of Radiation Oncology, Leuven, Belgium

**Keywords:** Hedgehog pathway, GLI transcription factors, GANT61, DNA repair, apoptosis

## Abstract

An essential role for Hedgehog (Hh) signaling in human cancer has been established beyond doubt. At present, targeting Hh signaling has mainly been investigated with SMO inhibitors. Unfortunately, resistance against currently used SMO inhibitors has already been observed in basal cell carcinoma (BCC) patients. Therefore, the use of Hh inhibitors targeting the signaling cascade more downstream of SMO could represent a more promising strategy. Furthermore, besides the classical canonical way of Hh signaling activation, non-canonical activation of the GLI transcription factors by multiple important signaling pathways (e.g. MAPK, PI3K, TGFβ) has also been described, pinpointing the importance of targeting the transcription factors GLI1/2. The most promising agent in this context is probably the GLI1/2 inhibitor GANT61 which has been investigated preclinically in numerous tumor types in the last few years. In this review, the emerging role of Hh signaling in cancer is critically evaluated focusing on the potential of targeting Hh signaling more downstream of SMO, i.e. at the level of the GLI transcription factors. Furthermore, the working mechanism and therapeutic potential of the most extensively studied GLI inhibitor in human cancer, i.e. GANT61, is discussed in detail. In conclusion, GANT61 appears to be highly effective against human cancer cells and in xenograft mouse models, targeting almost all of the classical hallmarks of cancer and could hence represent a promising treatment option for human cancer.

## INTRODUCTION

In this review, the emerging role of Hedgehog (Hh) signaling in cancer is critically evaluated focusing on the potential of targeting Hh signaling as an anticancer strategy. More specifically, the relevance of targeting Hh signaling more downstream of Smoothened (SMO), i.e. at the level of the glioma-associated oncogene homolog (GLI) transcription factors, is highlighted. Furthermore, the working mechanism and therapeutic potential of the most extensively studied GLI-inhibitor in human cancer, i.e. GANT61, is discussed in detail.

### The Hh signaling pathway

The Hh pathway, one of the major developmental pathways, is a very complex signaling network comprising both canonical and non-canonical signaling. Activation of canonical Hh signaling occurs when one of the ligands, i.e. Sonic (Shh), Desert (Dhh) or Indian (Ihh) Hedgehog binds to its receptor Patched (PTCH). This relieves the repression of SMO by PTCH and results in the accumulation of SMO in the primary cilium. Activated Smo in turn, facilitates the activation of the GLI transcription factors which will translocate to the nucleus and promote transcription of the Hh target genes. The GLI family of transcription factors consists of three different proteins (GLI1, GLI2 and GLI3), of which only GLI1 is an exclusively full-length transcriptional activator. GLI3 and, to a lesser extent, GLI2 can be partially processed into truncated repressor forms [1, 2]. The activation of the GLI transcription factors is controlled by Suppressor of Fused (SUFU) which is a key negative regulator of Hh signaling activity. In the absence of ligand binding, SUFU will directly bind the GLI proteins and inhibit their translocation to the nucleus and thus prevent pathway activation [3]. The anchorage of the GLI proteins in the cytoplasm by SUFU will facilitate processing and/or degradation of the GLI proteins and thereby inhibit Hh signaling [4]. To date, numerous target genes have been described, which are involved in feedback mechanisms (e.g. *HHIP, PTCH1, GLI1*), cell cycle regulation (e.g. *CYCLIN D1/2*), proliferation (e.g. *PDGFR, MYC*) apoptosis (e.g. *BCL2*), angiogenesis (e.g. *VEGF, ANG1/2*), epithelial-mesenchymal transition (EMT; e.g. *MMP9, SNAIL*) and self-renewal (e.g. *NANOG, SOX2*) [5, 6], representing a broad spectrum of mechanisms by which the Hh signaling pathway can be involved in carcinogenesis.

Hh signaling can also be activated by non-canonical signaling. Non-canonical Hh activation has been defined as either ligand-independent Hh activation originating from PTCH (Type I) and/or SMO (Type II), but independent of GLI-mediated transcription [7, 8] or through direct stimulation of the GLI transcription factors, independent of PTCH/SMO signaling [6, 9, 10]. Multiple important oncogenic pathways (e.g. PI3K, MAPK, Wnt, NF-κB and TGFβ) have been shown to activate Hh signaling. More specifically, PI3K, MAPK and TGFβ signaling induce their effect, at least partially, through the activation of the GLI1/2 transcription factors [11]. Moreover, crosstalk with tumor suppressor genes (e.g. P53, PTEN) has also been demonstrated [6, 9, 11], making this pathway a very interesting target for cancer therapy.

In recent years, the Hh signaling pathway has shown to be an essential key player in tumor initiation and/or progression to more advanced tumor stages [1, 2, 12, 13]. At the moment, inappropriate Hh signaling has been demonstrated in more than 30% of human cancers, including basal cell carcinoma (BCC), medulloblastoma (MB), melanoma, breast, prostate, lung, pancreatic, cervical and ovarian cancer [14]. Inappropriate Hh signaling has been ascribed to either ligand-dependent, i.e. autocrine and/or paracrine signaling, or ligand independent tumor cell intrinsic pathway activation due to loss-of-function mutations in *PTCH* or *SUFU* and gain-of-function mutations in *SHH, SMO* or *GLI1/2* [5, 15]. The latter has mainly been observed in BCC and MB.

### SMO inhibitors

The last decades, major progress has been made in the development of small molecules specifically inhibiting the Hh signaling pathway. Initially, pharmaceutical companies mainly focused on targeting SMO. Several SMO inhibitors are currently being tested in clinical trials for the treatment of multiple types of cancer. The most extensively studied SMO inhibitor is cyclopamine [16], a naturally occurring substance derived from the plant *Veratrum californicum,* which demonstrated high efficiency in preclinical studies, but failed clinical development due to poor pharmacokinetic characteristics (highly insoluble in water, poor chemical stability in acidic conditions), low potency and associated toxicity [17]. This led to the development of many small molecule Hh pathway modulators with improved potency and druggability, e.g. vismodegib (GDC-0449), IPI-926, sonidegib (LDE-225), BMS-833923, PF-04449913 and LY2940680. These SMO inhibitors seem to be highly efficient in patients with tumors harboring activating mutations in the Hh pathway, i.e. BCC and MB. In 2012, vismodegib has been approved for the treatment of advanced BCC on the basis of a phase II clinical trial with response rates of 30% and 43% in metastatic BCC and locally advanced BCC respectively [18]. Currently, sonidegib, BMS-833923 and IPI-926 have also proven efficacy in BCC and MB [19, 20]. At present, (clinical) investigations are ongoing to evaluate their efficacy in ligand-dependent Hh activated cancer types. However, these solid tumor types demonstrated little or no responsiveness in early phase clinical trials [21].

Regrettably, acquired resistance against vismodegib has already been reported in patients with advanced BCC and MB [22, 23]. In a study by Chang *et al.* evaluating re-growth in patients with BCC treated with continuous vismodegib, 6 out of 28 patients patients developed at least one tumor regrowth (mean time 56.4 weeks) while on the drug treatment [24]. Acquired resistance to SMO inhibition has been linked to distinct mechanisms, such as mutations in SMO (e.g., D473H) [25], amplification of the downstream transcription factor GLI2 [26] or up-regulation of synergistic signals such as PI3K signaling [27].

Possible solutions for these patients include (1) second-generation SMO inhibitors with a different mechanism of action that are still effective in vismodegib-resistant patients (e.g. HhAntag), (2) Hh pathway inhibitors more downstream of SMO (e.g. GANT58, GANT61) and (3) combination strategies with other molecular targeted therapies (e.g. PI3K, EGFR inhibitors), ionizing radiation or chemotherapy [13, 28].

The most promising targets within the Hh signaling pathway are by far the GLI transcription factors. First, these molecules are most downstream of the signaling pathway. Therefore, small molecules targeting the GLI transcription factors will still be effective in tumors harboring mutations in SMO or even more downstream of SMO (e.g. SUFU). Second, non-canonical activation of the GLI proteins occurs by several important oncogenic pathways. The fact that the GLI transcription factors are described as, at least partial, effectors of these oncogenic pathways highlights the potential therapeutic benefit of targeting these molecules.

## GLI1/2 AS EMERGING TARGETS FOR CANCER THERAPY

### Gli activation in tumors

The first indication of involvement of Hh signaling in cancer came from a study by Kinzler *et al.,* already in 1987, identifying a gene that was more than 50-fold amplified in malignant glioma. This gene was then named after the tumor, i.e. glioma-associated oncogene homolog 1 (GLI1) [29]. Currently, overexpression of GLI1 has been described in multiple other tumor types such as MB [30], rhabdomyosarcoma [31, 32], prostate [33, 34], biliary [35], breast [36-38], lung [39], colon [40, 41] and bladder [42] cancer. Moreover, higher GLI1 expression is associated with more advanced (and metastatic) tumors [33, 35, 38].

Several studies have also demonstrated the prognostic value of several Hh proteins in cancer patients. Protein expression of SHH, PTCH and GLI1 were all independent prognostic factors for both disease-free survival and overall survival in patients with colon cancer [40] and bladder cancer [42]. Fan *et al.* also demonstrated that low GLI1 expression correlates with a longer survival in patients with oral squamous cell carcinoma (SCC) [43]. Another study by ten Haaf *et al.* showed that GLI1 expression in breast cancer is associated with an unfavorable overall survival [37]. Furthermore, Chung *et al.* have observed that nuclear GLI1 expression is associated with metastasis and poor survival in patients with head-and-neck SCC after radiation treatment [44].

Since GLI1 is both a transcription factor and a target gene of Hh signaling, GLI1 expression is often considered as a measure for Hh signaling activity. Overexpression of GLI1 can be the result of either ligand-dependent or ligand-independent cell intrinsic Hh activation. Mutations at any level of the signaling pathway (e.g. PTCH1, SMO, SUFU) will result in an increased expression of GLI1. Amplification of the GLI transcription factors has only been described in a subset of tumor types, such as glioblastoma, BCC and bladder cancer [29, 45, 46]

### Targeting Hh signaling at the level of GLI1/2

Currently, only a few GLI inhibitors have been developed, whereas dozens of SMO inhibitors are on the market of which several are under clinical investigation. Both natural and synthetic GLI inhibitors have been described [47]. Triazole itraconazole is a natural anti-fungal agent that inhibits Hh signaling downstream of PTCH, but entails a different mechanism as known SMO inhibitors [48]. The mechanism of action of this compound is not completely understood, but is thought to be due to inhibition of GLI-mediated transcription [49]. Other natural compounds able to inhibit GLI transcription were identified in a large-scale screen for inhibitors of GLI transcription. Five compounds were shown to effectively inhibit both GLI1- and GLI2-mediated transcription, i.e. staurosporinone, zerumbone, arcyriaflavin C, physalin B and physalin F [50]. Several synthetic agents have been described to specifically target the GLI transcription factors, each with a different mode of action, e.g. HPI1-4, ATO, GANT58, GANT61, GlaB, JQ1 and I-BET151.

**Figure 1:**
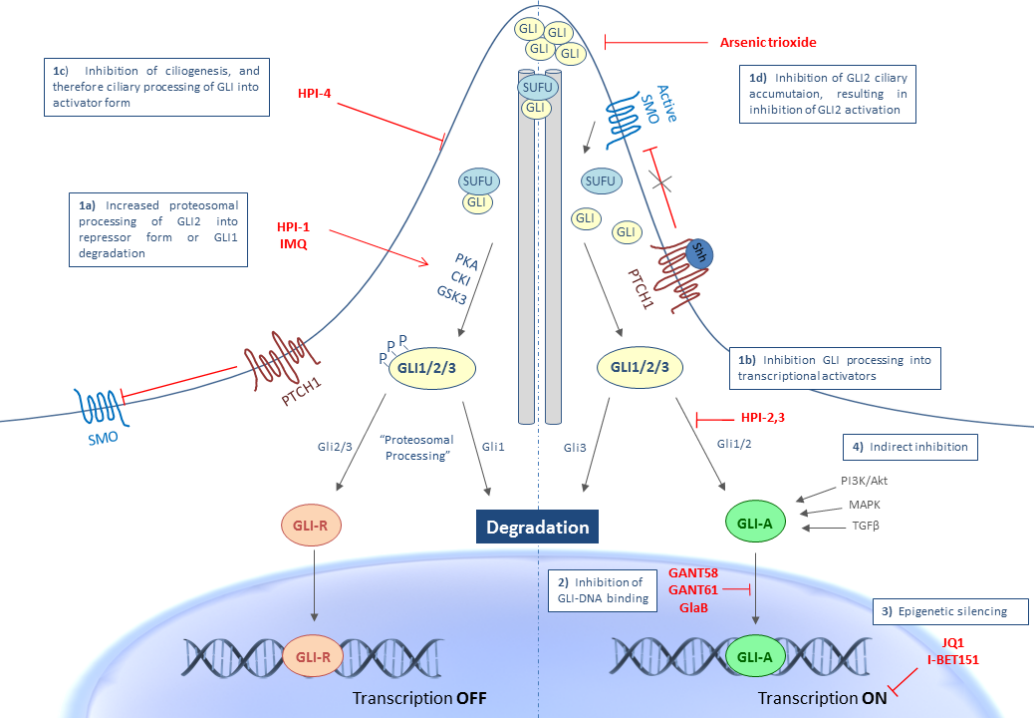
Targeting Hh signaling at the level of the GLI transcription factors GLI inhibition can occur at different levels in the activation process of GLI transcriptional output: **1a.** Increased proteosomal processing of GLI2 into repressor form or GLI1 degradation with HPI-1 or IMQ. **1b.** Inhibition of GLI processing into its activator form by HPI-2/3, **1c.** Inhibition of ciliogenesis and therefore processing into the activator form with HPI-4, **1d.** Inhibition of GLI2 ciliary accumulation and thus activation of of GLI2 by ATO. **2.** Inhibition at the level of GLI-DNA binding through GANT58, GANT61 or GlaB, **3.** Through epigenetic silencing with JQ1 or I-BET151 and **4**. Through indirect inhibition of non-canonical signaling pathways known to activate the GLI transcription factors. Abbreviations: CKI, casein kinase 1; GANT, Gli-ANTagonist; GlaB, Glabrescione; GLI, Glioma-associated oncogene homologue; GLI-A, activator form of GLI; GLI-R, repressor form of GLI; GSK3β, glycogen synthase kinase 3β; HPI, Hedgehog Pathway Inhibitor; IMQ, imiquimod; P, phosphate; PKA, protein kinase A; PTCH1, patched 1; SHH, sonic hedgehog; SMO, smoothened; SUFU, suppressor of fused.

Targeting Hh signaling at the level of GLI1/2 can be classified in four categories, as shown in Figure 1: 1) inhibition of GLI processing and its trafficking (post-translational modifications), 2) inhibition of GLI-DNA binding, 3) inhibition of transcriptional output, and 4) indirect inhibition.

#### Inhibition of GLI processing and trafficking

The primary cilium plays an essential role in the trafficking and posttranslational processing of the GLI transcription factors. Appropriate ciliary function is important for processing of both the repressor and activator forms of GLI proteins. As depicted in Figure 1, in the absence of Hh ligand, SMO is retained from the primary cilium and therefore not able to activate the GLI transcription factors. In this state, SUFU will bind the GLI transcription factors and retain the GLI proteins to the cytoplasm, thereby facilitating GLI processing. The sequential phosphorylation of the GLI proteins by protein kinase A (PKA), casein kinase 1 (CK1) and glycogen synthase kinase 3β (GSK3β) targets the proteins to the proteasome for (partial) degradation. GLI inhibition can occur at different levels in the activation process of the GLI transcription factors. Increased proteosomal processing to their repressor forms, decreased processing into transcriptional activators and reduced trafficking of the GLI proteins are several mechanisms that will result in inhibition of GLI transcriptional activity. Unlike GLI2 and GLI3, GLI1 is not cleaved to a repressor form, but will be degraded by the ubiquitin-proteasome system (UPS). In general, GLI1 protein levels are strictly controlled to allow proper target gene transcription and prevent inappropriate signaling activity. A study by Huntzicker et al has shown that degradation of the GLI1 proteins occurs quite rapidly and is controlled by two independent destruction signals (degron D_C_ and degron D_N_). Mutations in one or both degrons resulted in the accumulation of GLI1 proteins, which was significantly correlated with an increased transcriptional activity, thereby contributing to carcinogenesis [51]. Targeting the accumulation of the GLI proteins might therefore also represent a promising anticancer strategy.

A study by Hyman *et al.* describes four Hedgehog pathway inhibitors (HPIs) that are able to modulate GLI activity each with a unique mechanism of action. HPI-1 has been shown to increase the GLI repressor level, likely through posttranslational processing (PKA phosphorylation). HPI-2 and HPI-3 appeared to interfere with the processing of GLI2 to its transcriptional activator form. HPI-4 seemed to act on ciliogenesis, thereby inhibiting the processing of GLI into their activator form [52].

Recently, a novel role has been ascribed to the Toll-like receptor-7/8 (TLR7/8) agonist, imiquimod (IMQ), which has been approved for the treatment of BCC. IMQ negatively regulates Hh signaling in a PKA-dependent manner. More specifically, IMQ binds to adenosine receptors (ADORAs) which activate PKA, resulting in the phosphorylation and subsequent degradation of GLI1 [53, 54]. In this context, other molecules that stimulate ADORA/PKA signaling could represent a new class of anticancer therapy by repressing Hh signaling.

Arsenic trioxide (ATO), currently used for the treatment of acute promyelocytic leukemia (APL), has been demonstrated as a potent and specific inhibitor of Hh signaling. Kim *et al.* demonstrated that ATO inhibits GLI2 trafficking in and out the primary cilium, which is necessary for appropriate GLI2 activation. This ultimately results in a blockage of GLI2 accumulation in the primary cilium and subsequently destabilization of GLI2, which leads to a decreased level of GLI2 after long term treatment with ATO [55].

#### Inhibition through direct binding to GLI proteins

The GLI proteins belong to the family of zinc finger proteins, one of the most important families of DNA-binding proteins. The GLI protein consists of five zinc finger proteins of which only finger 1 does not make contact with the DNA. Zinc fingers 2 to 5 bind the major groove and wrap around the DNA helix. More specifically, zinc finger 2 and 3 are believed to mainly bind the DNA backbone whereas finger 4 and 5 directly make contact with the DNA base pairs [56, 57].

The first small molecules that were identified as Hh signaling inhibitors at the level of the GLI transcription factors were GANT58 and GANT61 (Gli-ANTagonist) [58]. Agyeman *et al.* have demonstrated that GANT61 directly binds GLI1 in a groove between zinc finger 2 and 3, which is not in the DNA binding region of GLI1, but this interaction does result in an inhibition of GLI-DNA binding and therefore GLI-mediated transcription [59]. The specific mechanism of action and therapeutic potential of GANT61 will be discussed in more detail below.

A recent study from Infante *et al.* describes a new GLI inhibitor Glabrescione B (GlaB) which also interferes with the interaction between GLI1 and DNA, but at the interface between zinc fingers 4 and 5, which are responsible for the interaction between GLI1-DNA [60].

#### Inhibition of Hh transcriptional output through epigenetic silencing

Two recent studies identified bromodomain and extra terminal (BET) proteins, and more specifically BRD4 proteins, as critical epigenetic regulators of Hh transcriptional output. BRD4 directly binds GLI1 and GLI2 promoters, thereby epigenetically regulating GLI transcription. In the first study, Tang *et al.* described the potential of JQ1, a BRD4 inhibitor, as an effective agent against Hh activity. Tumor growth was significantly attenuated in several tumor models with constitutive Hh pathway activation, even with resistance to SMO inhibitors [61]. In the second study, Long *et al.* performed a small molecule screen for epigenetic modulators against Hh signaling activity and identified the BET inhibitor, I-BET151 to effectively attenuate Hh activity, also through BRD4 inhibition. Treatment with I-BET151 also led to a decrease in tumor growth in an Hh-driven MB mouse model [62].

These studies provide evidence that targeting the BET bromodomain proteins, especially BRD4 inhibitors, could represent a promising future strategy to target Hh-driven tumors and could be effective in tumor cells harboring mutations in SMO or even more downstream of SMO. However, in addition to its antitumor effects, JQ1 has also shown to have an effect on several other important physiological and pathological processes like spermatogenesis, inflammation and cardiovascular disease [63]. Therefore, more investigation into the exact role of BRD4 inhibition in human cancer and potential toxicities due to multiple targeting is needed.

#### Indirect inhibition of GLI transcription factors

As stated above, the GLI transcription factors are modulated by several important oncogenic pathways, such as PI3K, TGFβ and MAPK signaling [10]. Consequently, targeted agents against these pathways could also be regarded as “indirect” inhibitors of Hh signaling.

Furthermore, one potential mechanism of resistance against SMO inhibitors has been ascribed to an increased activity of PI3K signaling. Simultaneous inhibition of both pathways has been demonstrated to significantly delay the development of resistance [27]. Moreover, a study by Wang *et al.* describes a link between mTOR signaling and GLI1 activity. Activated mTOR signaling resulted in an increased transcriptional activity and oncogenic function of GLI1. Simultaneous inhibition of mTOR (RAD-001) and Hh signaling (GDC-0449) resulted in an additional tumor growth inhibition *in vivo* in an esophageal xenograft mouse model compared to either single drug treatment [64].

## GANT61

In a cell-based screen for small molecule inhibitors of GLI-mediated transcription, Lauth *et al.* discovered GANT58 and GANT61 to selectively inhibit both GLI1 and GLI2-mediated gene transactivation. Both inhibitors caused significant inhibition of tumor growth, but GANT61 was shown to be more efficient [58]. This prompted further investigation of this agent and resulted in the publication of several preclinical studies performed in numerous cancer types, including rhabdomyosarcoma, neuroblastoma, leukemia, colon, pancreas, prostate, cervix, melanoma, lung, head-and-neck and gastric cancer. In this part of the review, we will summarize the findings of these studies, especially regarding the different target sites and therapeutic potential of GANT61 in human cancer to gain more insight into the exact working mechanisms of this agent.

The specificity of GANT61 to inhibit GLI-mediated transcription has been shown in multiple studies and, as stated above, was first described by Lauth *et al.* [58]. GANT61 has been demonstrated to decrease both gene and protein expression of the target genes GLI1 and PTCH1 and to reduce also the transcriptional output using GLI reporter assays in multiple cell types [58, 65-68].

Until recently, little was known about the exact working mechanism of GANT61. Then, Agyeman *et al.* investigated the mode of binding of GANT61 to the GLI transcription factors. By means of computational modeling, the authors showed that the biological activity of GANT61 is through direct binding to GLI1, in close proximity to, but independent of the DNA binding region of GLI1. GANT61 binds GLI1 in a groove between zinc finger 2 and 3 and has binding sites at amino acids E119 (1H bond) and E167 (2H bonds). Mutations in these two binding sites resulted in a significant inhibition of binding between GANT61 and GLI1, confirming the interaction between both molecules. Moreover, most of the amino acid residues within 3.5Ǻ of GANT61 appeared to be conserved between GLI1 and GLI2, which could explain the inhibitory effect of GANT61 also on GLI2-mediated transcription [59].

The cytotoxic effect of GANT61 has been investigated in numerous cancer cell types, with IC_50_ values ranging from 5μM-15μM after 48h-72h in most cancer cell lines (Table 1). In cell lines known to be independent of GLI signaling, higher concentrations ranging up to 90μM were needed to cause any significant cytotoxicity, probably rather due to nonspecific toxicity of the high dose of drug or diluent (i.e. ethanol or dimethylsulfoxide (DMSO)).

**Table 1:** Overview of IC_50_ values of GANT61 in different tumor types

Tumor type	Cell line	IC_50_ value	Treatment duration	Assay	Reference
MEF	NIH 3T3	5μM	48h	GLI reporter assay	58
Colon	HT29 HCT116 SW480 VRC5/c1 GC3/c1	5-15μM	72h	Clonogenic assay	81
Neuroblastoma	SK-N-AS SK-N-DZ IMR-32 SK-NBE(2) SH-SY5Y SK-N-FI	5,28-12,4μM	72h	Fluorometric microculturecytotoxicity assay (FMCA)	67
Cervix	HaCaT SiHa CasKi HeLa	5-10μM	48h	BrdU ELISA kit	94
Myeloid leukemia	Kasumi-1 K562 HL60 U937	<10μM – 75μM	48h	WST assay	107
Pancreas	Primary tumor	10μM/5μM	48h/72h	XTT assay	68
Lung	NCI-520 NCI-H226 SK-MES-1 NCI-H2170 (GLI-negative)	5-10μM No effect (>90μM)	96h	ApoLive-Glo Multiplex assay	79
Pleural mesothelium	H2452 H2373 H513 H2052 Gardner Gates H290 REN	2.5-10μM	96h	MTT assay	65
Biliary tract	GBC CCSW-1 MzChA-1	<10μM	72h	Resazurin test	35
Rhabdomyosarcoma	RH30 CW9019 SMS-CTR RD A204	100-250μM	24h	MTT assay	77

### Working mechanisms of GANT61 in human cancer cells

**Figure 2:**
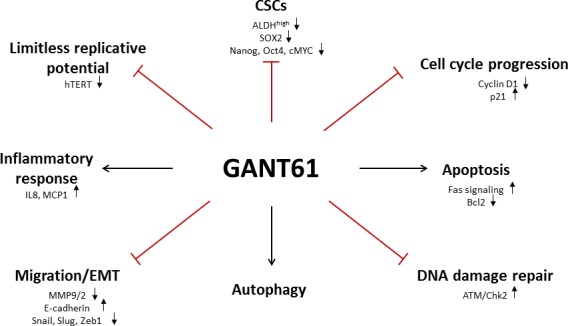
Schematic overview of different GANT61 target sites Inhibition of the GLI transcription factors with GANT61 targets many of the “classical hallmarks of cancer”, such as cell viability, proliferation, apoptosis, DNA damage repair, epithelial-mesenchymal transition (EMT), autophagy, cancer stem cells and immune response.

The next part of this review summarizes the main effects of GANT61 treatment, illustrating that GANT61 targets many of the “classical hallmarks of cancer”, such as cell viability, proliferation, apoptosis, DNA damage repair, epithelial-mesenchymal transition (EMT), autophagy, cancer stem cells and immune response (Figure 2).

#### Limitless replicative potential

An unlimited replicative potential is one of the most important hallmarks of cancer. Normal somatic cells have a limited potential to replicate due to the shortening of the telomeres, which are heterochromatic structures located at the ends of the chromosome and mainly function to protect the chromosomes from recombination, degradation and end-to-end fusion [69]. With every DNA replication cycle, the telomeres are shortened since DNA polymerase is unable to fully copy the ends of telomeric DNA in the absence of a template strand (‘end-replication effect’). When the telomeres become critically shortened, they fail to protect the chromosomal ends resulting in irreversible growth arrest and replicative senescence. Telomerase prohibits this telomere shortening by catalyzing *de novo* synthesis of telomeric DNA after cell division and aberrant activation of telomerase has been implicated in carcinogenesis [70]. The expression level of human telomerase reverse transcriptase (*hTERT*), a catalytic subunit bearing the enzymatic activity of telomerase, is considered the rate-limiting determinant of human telomerase activity [71]. Many factors have been implicated in the regulation of *hTERT* in cancer and normal cells, including Wnt signaling, c-Myc, HIF-1 and p53 [72-75]. In a recent paper, Mazumdar *et al.* have shown that Hh signaling transcriptionally regulates hTERT in colon, prostate and brain cancer cells, but not in the non-malignant 293T cells. Inhibition with GANT61 reduced hTERT protein and mRNA expression by preventing the binding of GLI1/2 with the hTERT promotor in human colon cells [76]. Thus, GANT61 can decrease the proliferative potential of cancer cells through interference with hTERT activity. Another mechanism by which GANT61 inhibits proliferation is by its effects on cell cycle progression. Several independent studies have shown that GANT61 induces a G1 arrest, consistent with decreased protein levels of the Hh target gene Cyclin D, which is a driver for the progression from G1 to S phase [67, 77, 78]. Moreover, GANT61 induced the expression of p21, which also inhibits cell cycle progression [76, 77].

#### Apoptosis

Cytotoxicity of GANT61 has frequently been associated with increased cell death rather than a direct effect on cell proliferation. Inhibition of Hh signaling can cause apoptosis either through activation of Fas signaling or through decreasing protein levels of the anti-apoptotic Bcl2, which is one of the target genes of Hh signaling. Multiple independent studies have demonstrated that GANT61 induces cell death through both mechanisms. On the one hand, GANT61 induced Fas signaling, characterized by increased protein levels of Fas, cleaved caspase 3, cleaved PARP [67, 79, 80] and death receptor 5 (DR5). PDGFRα levels were decreased after GANT61, which potentially contributed to the increase of Fas-mediated apoptosis, since PDGFRα regulates Fas expression [68, 81]. On the other hand, GANT61 also decreased protein levels of Bcl2, contributing to its extensive apoptotic effect in cancer cells [68, 81]. Moreover, overexpression of a double-negative FADD (Fas-associated death domain) protein to abrogate Fas/DR5-mediated death receptor signaling and/or Bcl2 partially rescued the GANT61-induced cytotoxicity. This indicates that GANT61-induced cytotoxicity can be, at least partially, ascribed to effects on cell death.

A recent study by Lim *et al.* described a novel mechanism for GANT61-induced apoptosis in malignant mesothelioma cells. Induction of apoptosis by GANT61 was shown to be dependent on the production of mitochondrial reactive oxygen species (ROS), but independent of GLI1 or GLI2. Moreover, down-regulation of GLI1, GLI2 and PTCH gene levels by GANT61 was also shown to be mediated by ROS and could be counteracted by the addition of anti-oxidants, indicating that GANT61 at least partially acts through the induction of oxidative stress [82].

#### DNA damage repair

GANT61-induced cytotoxicity can also be ascribed to its inhibitory effect on DNA damage repair. Mazumdar *et al.* demonstrated that GANT61 was able to induce DNA double strand breaks (DSBs) marked by γH2AX, an ATM-dependent DNA damage response mechanism. Activation of ATM (p-ATM) and Chk2 (p-Chk2) was already shown 4h after incubation. The latter resulted in the induction of DSBs and ultimately led to the induction of apoptosis after 24h-48h. No effect of GANT61 was observed on the ATR/Chk1 axis [83]. In addition, Shi and colleagues performed a cDNA microarray of 18,401 genes to identify differentially expressed genes after GANT61 treatment in two colon cancer cell lines, HT29 and GC3/c1 cells. Gene expression of several molecules involved in the DNA damage repair was significantly decreased after GANT61 treatment [84].

#### Migration

Hh signaling is known to be implicated in epithelial-mesenchymal transition (EMT) and therefore also in the initiation of metastasis. Fan *et al.* demonstrated upregulation of SHH, GLI1 and MMP9 and down-regulation of E-cadherin in oral SCC tissue compared to normal tissue [43]. In addition, a negative correlation between GLI1 and E-cadherin was described in several studies [43, 85, 86]. Contrary, Joost *et al.* have shown that low GLI1 levels promote EMT in pancreatic ductal adenocarcinoma cells. Moreover, they also demonstrated that GLI1 directly regulates E-cadherin levels through binding with its promotor (CDH1) [87]. Chen *et al.* found that downregulation of GLI1 expression significantly suppressed adhesion, motility, migration, and invasion of hepatocellular carcinoma cells, which was correlated with reduced expression of MMP2 and MMP9, upregulation of E-cadherin, and concomitant down-regulation of Snail and Vimentin; all consistent with EMT inhibition [86].

Non-canonical Hh signaling has also been implicated in EMT regulation. Xu *et al.* describe an EMT molecular network mediated by Hh signaling in pancreatic cancer cells. Their data show that GLI1 signaling promotes EMT by inducing a complex signaling network including TGFβ, PI3K, RAS and Wnt signaling [88]. Also, Ke *et al.* demonstrated that GLI1 promotes EMT, invasion and migration in ovarian cancer cells through crosstalk with PI3K signaling [89]. A recent study by Li *et al.* has shown that GLI1 regulates TGFβ-induced EMT in non-small cell lung cancer cells. Inhibition of GLI1 with GANT61 attenuated induction of EMT by TGFβ [90]. Furthermore, another study indicated that invasion and EMT in pancreatic cancer cells is regulated by SDF-1/CXCR4 signaling, which non-canonically activates Hh signaling in a SMO-dependent manner. Inhibition of Hh signaling through cyclopamine or GLI silencing blocked this SDF-1/CXCR4-mediated invasiveness [91].

Hypoxia also plays an important role in EMT and invasion. Work from Lei *et al.* has shown this is, at least partially, through activation of GLI1 by hypoxia. Knockdown of GLI1 did not have an effect on hypoxia-induced HIF1α expression, but completely eliminated the hypoxia-induced vimentin and E-cadherin expression and tumor cell invasiveness [92, 93].

The effect of GANT61 on migration and invasion has been investigated in multiple studies. As expected, GANT61 has been shown to slow down cell migration and thus to decrease cell motility [78, 80, 94]. Furthermore, Fu and colleagues have also demonstrated that this is correlated with a decreased expression of the EMT markers Snail, Slug and Zeb1 in pancreatic cancer cells [68]. Inhibition of GLI signaling by means of GANT61 could hence be a promising target to decrease tumor cell motility and invasiveness.

#### Cancer stem cells

Several studies have indicated that Hh signaling plays a key role in the regulation of cancer stem cells (CSCs), by controlling the transcription of a number of genes implicated in cell fate determination and stemness features, i.e. self-renewal and pluripotency [68, 95-97]. Work of Santini *et al.* has shown that melanomas contain a subpopulation of cells expressing high ALDH activity (ALDH^high^), which is correlated with a higher ability to self-renew and tumorigenicity. GANT61 significantly reduced the number and self-renewal capacity of these melanoma CSCs and also decreased tumor initiation *in vivo* [96]. Similarly, a study by Heiden *et al.* showed that inhibition of GLI1 significantly reduced the number of ALDH^high^ thyroid CSCs. Overexpression of GLI1 on the other hand increased the number and the self-renewal of these cells [97]. Furthermore, silencing of SOX2 in melanoma CSCs also decreased self-renewal *in vitro* and limited *in vivo* tumor initiation and growth of melanoma stem cells. Hh signaling directly regulates SOX2 transcription through direct binding of the GLI transcription factors in the promotor region of SOX2, indicating that the effect of GANT61 is at least partially mediated by SOX2 [98]. This is in line with another study by Fu *et al.* in which the authors have demonstrated that GANT61 significantly decreases protein levels of several markers of self-renewal such as SOX2, NANOG, OCT4 and cMYC. Moreover, GANT61 inhibited pancreatic CSC tumor growth in NOD/SCID IL2Rγ null mice [68].

It is well known that CSCs are more resistant to chemo- and radiotherapy [99, 100]. The use of Hh inhibition in combination with chemotherapeutics could be a promising strategy. Indeed, GANT61 has been shown to potentiate the effect of chemotherapeutics in neuroblastoma cells [67] and biliary tract cancer cells [35] in an additive and/or synergistic manner. In a model of alternating therapies proposed by Blagoskonny [101], Hh-dependent cancer cells could first be targeted with Hh inhibitors. Over time, acquired resistance to these drugs may occur, as has already been observed with the use of vismodegib in BCC patients. Since the relapsed tumor will still be dependent on universally-vital targets, it can then be targeted with chemotherapeutics. As chemotherapy preferentially kills proliferating cells, it may spare CSC that are driven by embryonic/stem pathways such as the Hh signaling. Moreover, resistance against chemotherapy has often been associated with an upregulation of developmental pathways, such as Hh, Wnt and Notch signaling [102]. Therefore, once chemoresistance has occurred, the relapsed tumor might be sensitive to Hh inhibitors again. Next to that, GANT61 could also be an attractive target to sensitize radioresistant CSCs to radiation treatment, as it has been shown that knockdown of SHH or GLI significantly reduced clonogenic survival, while expression of GLI1 was correlated with the number of surviving colonies after ionizing radiation [97].

#### Autophagy

Several studies have shown that GANT61 induces autophagy, contributing to decreased cell viability and increased apoptosis. Inhibition of autophagy decreased the GANT61-induced apoptosis both *in vitro* and *in vivo* in hepatocellular and pancreatic cancer cells, highlighting the role of autophagy in GANT61-induced cytotoxicity [103, 104].

#### Immune response

Little is known about the role of Hh signaling in the immune response. A study by Yoshimoto *et al.* illustrated that GANT61 increased the production of the inflammatory cytokines IL8 and MCP1, thereby increasing monocyte recruitment in CT26 colon cancer cells. Activation of Hh signaling appeared thus to be associated with an anti-inflammatory effect in colon cancer cells [105].

### Therapeutic potential of GANT61 in animal models

Besides the promising results of GANT61 in *in vitro* studies, also several animal studies have shown significant decreases in tumor growth upon GANT61 treatment (Table 2) [58, 67, 68, 77, 79, 104, 106]. Lauth *et al.* even reported complete tumor regression in a 22Rv1 prostate cancer xenograft mouse model. Treatment with GANT61 significantly decreased BrdU incorporation and increased apoptosis in these tumor compared to controls [58].

**Table 2:** Overview of GANT61 treatment in animal models

Tumor type	Animal model	Dose	Formulation	Treatment duration	Efficacy	Ref.
Prostate	22Rv1 xenograft (BALB/c nude (nu/nu) mice)	50mg/kg	Every other day (s.c. injection)	16 days	Tumor regression until no tumor palpable	58
Neuroblastoma	SK-N-AS xenograft (NMRI-nu/nu mice)	50mg/kg	Daily (gavage)	12 days	Tumor growth reduction (63% of control)	67
Pancreas	Pancreatic CSC xenograft (humanized NOD/SCID IL2γ null mice)	40mg/kg	3 times/week (i.p. injection)	6 weeks	CSC tumor growth inhibition	68
Hepatocellular	Huh7 xenograft (SCID mice)	50mg/kg	Every other day (i.p. injection)	4 weeks	Tumor growth reduction	104
Lung	NCI-H520, NCI-H2170 and NCI-H226 xenograft (immune deficient Rag1^−/−^ mice)	50mg/kg	Every other day (i.p. injection)	20 days	Tumor growth reduction	79
Rhabdomysarcoma	RD, RH30 xenograft (athymic nu/nu mice)	50mg/kg	3 times/week (i.p. injection)	Until tumor size control mice >1cm^3^ (21-33 days)	Tumor growth reduction (53% in RD and 47% in RH30 cells compared to control)	77
Embryonal rhabdomyosarcoma	CCA, Rh36 and A673 cells introduced in Chick chorioallantantoic membrane (CAM) assay	10μM /30μM	Pre-mixture of cells with GANT61	7 days	Decreased tumor volume	106

### Combined modality treatment

As already indicated for a few times, the Hh signaling interacts with multiple signaling pathways at several levels of the signaling cascade. Most important pathways are the RAS/RAF/MEK/ERK, PI3K/AKT/mTOR, epidermal growth factor receptor (EGFR) and Notch signaling, which all interact at the level of the GLI transcription factors, except for Notch which interferes with the ligand SHH. Combined treatment modalities could be useful to overcome or delay resistance frequently observed after long-term treatment with a single agent such as vismodegib or tyrosine kinase inhibitors. Especially the combination of Hh and PI3K signaling inhibition could be beneficial, since one potential mechanism of resistance against SMO inhibition has been ascribed to upregulation of PI3K signaling. Combination of a PI3K inhibitor (BKM120 or BEZ235) and SMO inhibitor (LDE225) delayed the development of resistance in a MB mouse model, although no effect was observed on tumor growth [27]. This combination is currently under clinical investigation in patients with advanced or metastatic BCC (NCT02303041) and patients with advanced solid tumors (NCT01576666).

Another promising combination strategy could be the combination of an mTOR inhibitor and GANT61. Two independent studies have already shown that this combination is significantly more effective than either single treatment. In the first study, Pan *et al.* have demonstrated a synergistic effect of rapamycin and GANT61 in myeloid leukemia cells [107]. The second study by Srivastava *et al.* indicated that GANT61-mediated cytotoxicity was significantly more pronounced in combination with mTOR inhibitors emsirolimus and rapamycin, but also in combination with the chemotherapeutic agent vincristine in rhabdomyosarcoma cells [77].

As mentioned earlier, combination of Hh inhibition with radiotherapy might also be a promising strategy to increase the sensitiveness of certain radioresistant types of cancer. A very recent study by Zhou et al investigated the role of GLI1 in radioresistance and found that GANT61 could increase radiosensitivity of renal cell carcinoma cells through hampering of DNA damage repair. Moreover, simultaneously targeting of GLI1 and HIF2α turned out to be even more effective [108].

## CONCLUSIONS AND FUTURE PERSPECTIVES

In recent years, the Hh signaling pathway has proven to be an essential key player in tumor initiation and/or progression to more advanced tumor stages. Numerous SMO inhibitors are currently on the market of which several are under clinical investigation. Initially, SMO inhibitors seemed to be very efficient in ligand-independent tumor types, such as BCC and MB, but unfortunately resistance against these agents has already been observed. Moreover, the effect of SMO inhibitors in other (ligand-dependent) types of cancer seems to be very limited, highlighting the importance of identifying new target sites. The most promising targets within the Hh signaling pathway are by far the GLI transcription factors for several reasons. First, these molecules are most downstream of the signaling pathway, meaning that small molecules targeting the GLI transcription factors will still be effective in tumors harboring mutations in SMO or even more downstream of SMO (e.g. SUFU). Additionally, non-canonical activation of the GLI transcription factors occurs by several important oncogenic pathways, which would render these tumors insensitive to inhibitors more upstream of the Hh signaling cascade. With this review, we attempted to highlight the importance of targeting Hh signaling more downstream of SMO, more specifically, at the level of the GLI transcription factors. In the second part of this review, we summarized currently published data on the GLI1/2 inhibitor GANT61, underlining its efficacy and the different mechanisms by which this agent interacts with cancer cells. In conclusion, GANT61 appears to be highly effective against human cancer cells and in xenograft mouse models, targeting almost all of the classical hallmarks of cancer and could hence represent a promising treatment option for human cancer. However, the impact of GLI inhibition on other important cancer mechanisms such as angiogenesis has not yet been investigated and should be examined, since this would have major implications for the future use of GANT61. Additionally, at the moment there is little/no knowledge on the pharmacokinetics (e.g. solubility, metabolism, etc.) of this agent and its toxicity.
